# Recent advances in understanding tight junctions

**DOI:** 10.12703/r/10-18

**Published:** 2021-02-23

**Authors:** Mikio Furuse, Yoshimi Takai

**Affiliations:** 1 Division of Cell Structure, National Institute for Physiological Sciences, Okazaki, Aichi, Japan; 2 Department of Physiological Sciences, School of Life Science, SOKENDAI, The Graduate University for Advanced Studies, Okazaki, Aichi, Japan; 3 Division of Pathogenetic Signaling, Department of Biochemistry and Molecular Biology, Kobe University Graduate School of Medicine, Kobe, Japan

**Keywords:** tight junction

## Abstract

Tight junctions (TJs) are one type of cell–cell junction in epithelial cell types in vertebrates. They form a paracellular diffusion barrier and create the boundary between the apical and basolateral plasma membrane domains. The molecular constituents of TJs have mostly been identified, and now their cell biology has shifted to understanding of their formation, dynamics, and functional regulation as well as their relationship to the organization of epithelial cells. Accumulating novel findings are supported by new methods, including super-resolution microscopy, quantitative microscopy, biophysical measurements, and genome editing-mediated gene manipulation. As a conceptual breakthrough, liquid-liquid phase separation seems to be involved in the formation of TJs as super-molecular complexes. This short article summarizes seminal studies in the cell biology of TJs from the last three years.

## Molecular structure of claudin-based tight junction strands

The functional unit of tight junctions (TJs) is a TJ strand, which is a fibril-like polymer of claudin molecules within the plasma membrane. Based on their crystallization of a single row of claudin-15, one of the channel-type claudins, Fujiyoshi’s lab proposed that TJ strands consist of claudin molecules that assemble into antiparallel double rows^1,2^. However, conventional freeze-fracture replica electron microscopy could not visualize such a feature of TJ strands possibly because of the limitation of spatial resolution due to platinum crystals. To overcome this problem, Krystofiak *et al*. combined amorphous carbon replicas with phase-contrast electron microscopy and found that TJ strands have double-stranded morphology^3^. This observation supports the antiparallel double row model. Using Fujiyoshi’s model, Samanta *et al*. further examined the structure of the claudin-15 channel using molecular dynamics simulation and physiological measurements of the channel property of claudin-15 mutants^4^. According to the simulation, claudin-15 forms paracellular pores with a mean radius of 4.2 Å in the narrowest region, which allows the passage of small dehydrated ions such as Na^+^ but can discriminate larger ions. Furthermore, the authors proposed a detailed model for a potential three-dimensional organization of the selectivity filter of the mouse claudin-15 channel. Among three negatively charged amino acids in the first extracellular loop, Glu46, Asp55, and Asp64, the simulation predicted that Asp55 has a key role in regulating the charge selectivity for the monovalent cation, while Glu46 and Asp64 have relatively minor roles. This notion was corroborated by the following physiological measurement of epithelial cells expressing wild-type or mutant claudin-15^4^.

## Dynamics of TJ strands

Among two modes of the paracellular pathway, the leak pathway for slow passage of solutes containing macromolecules is thought to need dynamic reorganization of TJ strands, including their breaking and annealing. Using a zinc-binding dye, Stefanson *et al*. have developed a new method for live imaging of the disruption of TJ barriers with high spatial and temporal resolution under a fluorescence microscope^5^. By applying this technique to the epithelium of *Xenopus* embryos, they detected sporadic break of TJ barrier and the following repair. Interestingly, the break of the TJ barrier caused by local reduction of TJ proteins induced recruitment of active Rho at the sites, followed by reinforcement of actin filament (F-actin), myosin II, and TJ proteins (so-called Rho flares). Rho activation, F-actin polymerization, and ROCK-mediated myosin II activation were all required for TJ recovery, suggesting a role for actomyosin-mediated contraction in this process.

Live imaging of the dynamics of individual TJ strands in epithelial cells, which may be the final goal of TJ imaging, has not been reported yet, even in super-resolution fluorescence microscopy. As a preliminary step of this, reconstituted TJ strand-like structures generated in fibroblasts were observed by fluorescence live cell imaging^6^. Van Itallie *et al*. further performed super-resolution live imaging of TJ-strand-like structures generated by N-terminal GFP-tagged claudin-2, which could bind to a scaffolding protein ZO-1, in Rat-1 fibroblasts^7^. The dynamics of a patch of claudin-2 strand network was constrained by ZO-1 and influenced by F-actin through ZO-1. Co-expression of occludin never changed claudin-2 strand dynamics, but occludin colocalized with claudin-2 at strand ends and junctions. Claudin-2 strands often showed breaking and annealing at TJs regardless of ZO-1 interaction. Pulse-chase-pulse experiments of SNAP-tagged claudin-2 strands revealed that newly synthesized claudin-2 is added to strand ends and TJs. In a subsequent study, Van Itallie *et al*. also showed in MDCK cells that newly synthesized claudins are added to the basal side of TJs, where free ends of TJ strands are often seen, consistent with the observation in the Rat-1 model system^8^.

Regarding the basic mechanism of TJ formation in epithelial cells, Shigetomi *et al*. analyzed α-catenin-deficient mouse EpH4 epithelial cells, which lack adherens junctions (AJs) and TJs^9^. Cholesterol level was reduced in the α-catenin-deficient EpH4 cells, and addition of cholesterol restored TJ strand formation. These results provide a new idea that AJs regulate TJ formation via cholesterol contents and suggest the involvement of membrane microdomains in TJ formation. This study also showed that the mechanical stabilization of cell contacts via AJs is not necessarily required for TJ formation in epithelial cells^9^.

## ZO-1 revisited: actomyosin regulation, mechanosensation, and phase separation

ZO-1 is a TJ scaffolding protein with multiple domains for protein–protein interactions. In addition to critical roles for ZO-1 and a related protein, ZO-2, in TJ formation, ZO-1 is known to be involved in AJ formation and actomyosin regulation. Odenwald *et al*. found that the apical brush border structure is aberrant in ZO-1-deficient intestinal epithelial cells in mice^10^. ZO-1-knockdown MDCK cells also showed abnormal apical architecture with remarkable changes in subapical F-actin. Interestingly, the U5-GuK domains of ZO-1, but not its actin-binding region, was responsible for the proper apical architecture. The apical organization impairment by ZO-1 depletion was normalized by myosin II inhibition. Consistently, the elevation of the apical epithelial tension was measured by Cartagena-Rivera *et al*. using noncontact acoustic frequency-modulation atomic force microscopy^11^. Otani *et al*. generated ZO-1/ZO-2 double knockout MDCK cells using genome editing^12^. Because these cells had complete deficiency of ZO-1 and ZO-2, they showed abnormalities in epithelial architecture much more remarkably than previously reported ZO-1/ZO-2 double knockdown cells^10^, including severe disruption of apical cell–cell junctions, mislocalization of the aPKC polarity complex, impairment of apical-basal plasma membrane polarity, and aberrant actomyosin contraction^12^. These studies imply that ZO-1 acts as more than a simple TJ scaffold; it is also a key factor for the organization of epithelial cells at least by regulating actomyosin function. This appears to be consistent with the previous observation by Fanning *et al*. that ZO-1/ZO-2-depleted MDCK cells by RNAi showed accumulation of junctional actomyosin with recruitment of phospho-myosin light chain, contraction of the actomyosin ring, and expansion of the apical domain, suggesting a role for ZO-1/ZO-2 in the apical organization of epithelial cells via actomyosin regulation^13^.

On the other hand, ZO-1 appears to be controlled by actomyosin contraction. Spadaro *et al*. generated ZO-1 tagged with two different epitopes at its N- and C-terminus, respectively, and introduced it to ZO-1-knockout EpH4 cells^14^. Super-resolution microscopy revealed that the signals from these two epitope tags are spatially separated, indicating stretched conformation of ZO-1 with regular arrangement. Myosin inhibition canceled the separation of these two epitopes, suggesting that ZO-1 stretching is actomyosin tension dependent. Furthermore, mechanical stretching of a single ZO-1 molecule using magnetic tweezers revealed its stepwise unfolding events. The group proposed that tension stretches ZO-1, followed by recruitment of its ligands to activate downstream signaling^14^. Haas *et al*. generated a fluorescence resonance energy transfer-based tension sensor of ZO-1 and confirmed that ZO-1 is indeed under mechanical tension in epithelial cells^15^. They further found that the mechanical load on ZO-1 increases with extracellular matrix stiffness and that JAM-A, an Ig domain-containing TJ membrane protein, controls the load on TJs by inhibiting the recruitment of Rho and ROCK.

Belardi *et al*. investigated the role of ZO-1 binding to F-actin in TJ function. They identified a 28-amino-acid actin-binding site (ABS) in the actin-binding region of ZO-1 in its C-terminal half. A ZO-1 mutant lacking the ABS did not fully rescue epithelial barrier function when introduced into ZO-1/ZO-2 double knockout cells regardless of its localization at cell–cell contacts and recruitment of other TJ proteins. By comparing the ability to recover epithelial barrier function among ZO-1 mutants in which the ABS was replaced with actin-binding domains of other proteins, the group concluded that a weak affinity of ZO-1 with F-actin is the key to generate robust TJ barrier with a sufficient amount of TJ structure^16^. Interestingly, the ZO-1 mutants that strongly bind to F-actin provided leaky TJs, suggesting that tunable interaction of ZO-1 with F-actin is important for proper TJ barrier formation.

One of the current topics in cell biology is the roles of liquid-liquid phase separation (LLPS) in the formation of membraneless organelles and dynamic cellular processes^17^. Beutel *et al*. discovered that ZO-1, ZO-2, and ZO-3 form condensed compartments by LLPS^18^. Fluorescent protein-tagged ZO proteins transiently overexpressed in MDCK cells or HEK293 cells showed drop-like non-junctional assemblies, and fluorescence recovery after photo bleaching analyses confirmed their liquid-like properties. The study using purified ZO proteins demonstrated their LLPS *in vitro*. Domain analyses of ZO-1 deletion mutants revealed that the PDZ-SH3-GUK supra-domain is required for LLPS, while the following U6 domain acts as a negative regulator, probably via auto-inhibition. LLPS properties of ZO-1 mutants correlated with the ability of enrichment in cell–cell junctions and TJ formation in ZO-1/ZO-2-deleted MDCK cells. The group showed that binding to other proteins, de-/phosphorylation, or mechanical force activates ZO-1 for LLPS from self-inhibition^18^. LLPS of ZO-1 was also reported in gastrulating zebrafish embryos by Schwayer *et al*.^19^. At TJs formed between the enveloping cell layer (EVL) and the yolk syncytial layer (YSL), ZO-1 accumulation was actomyosin contractility dependent. Live imaging of transgenic zebrafish expressing fluorescent protein-tagged ZO-1 showed non-junctional clusters of ZO-1 in YSL, and they were incorporated into TJs between the EVL and YSL. Analyses of the behavior of the ZO-1 clusters supported the finding that they are formed by LLPS.

## New perspective of the barrier and fence function of TJs

Otani *et al*. generated claudin quintuple-knockout MDCK cells, which completely lacked TJ strands^12^. Unexpectedly, these cells still had close plasma membrane appositions with a barrier function to the passage of macromolecules, which were mediated by JAM-A. A novel idea was proposed that the TJ-mediated paracellular barrier consists of a claudin-mediated barrier to small molecules and a JAM-A-mediated barrier to macromolecules.

In addition to their role as a paracellular diffusion barrier, TJs have been thought to act as a fence that hampers lateral diffusion of membrane proteins and lipids between the apical and basolateral membrane domains. However, how TJs create the fence still remains elusive. As a bottom-up approach, Belardi *et al*. reconstituted claudin-4 into giant unilamellar vesicles (GUVs) in an oriented manner using a microfluidic jetting technique^20^. GFP-tagged claudin-4 containing GUVs assembled and claudin-4 accumulated at the GUV–GUV interface, although TJ strand formation was not examined. The outer leaflet of the lipid bilayer at this interface permitted penetration of phospholipid molecules labeled with a fluorescent dye of ~600 Da but excluded those conjugated with a protein tag of 5 nm height. This physical segregation by steric hindrance may work as a fence at TJs against lateral diffusion of integral membrane proteins between apical and basolateral plasma membrane domains. Interestingly, claudin quintuple-knockout MDCK cells had normal plasma membrane polarity despite the absence of TJ strands, which had been believed to work as a molecular fence for a long time^12^. To explain this paradox, several possibilities for their combination can be considered^21^. A belt of JAM-A-mediated close membrane attachment at apical junctions in claudin quintuple-knockout MDCK cells may exclude membrane proteins in a size-dependent manner by steric hindrance. Alternatively, the clustering of JAM-A may induce molecular crowding, which works as a fence against the lateral diffusion of membrane proteins and lipids. It is also possible that the JAM-A clustering at cell–cell contacts generates lipid microdomains, which exclude other molecules. These possibilities need to be investigated further.

## Claudin-mediated intracellular signaling

Besides the roles of claudins in TJ formation and the regulation of epithelial barrier function, recent studies reported their involvement in intracellular signaling that influences cellular behavior, including proliferation, differentiation, and migration. Sugimoto *et al*. previously showed that exogenous expression of human claudin-6 in mouse F9 embryonic carcinoma cells triggered epithelial differentiation^22^. They further investigated this phenomenon and found that Src-family kinase activation occurs in claudin-6-expressing F9 cells dependent on the second extracellular loop and two conserved tyrosine residues, Tyr196 and Tyr200, in the COOH-terminal cytoplasmic region of claudin-6^23^. The research group showed that Src-family kinase signaling finally targets the retinoic receptor via the PI3 kinase/AKT axis and proposed a claudin-mediated signal transduction that transmits cell adhesion signaling to the nucleus. Li *et al*. found that collective cell migration behavior of human SAS squamous cell carcinoma cells depends on claudin-11 expression^24^. They showed that claudin-11 suppressed RhoA activity at cell–cell contacts by recruiting p190RhoGAP, whose activation was regulated by Src-mediated phosphorylation. Two tyrosine residues, Tyr191 and Tyr192, in the COOH-terminal cytoplasmic region of claudin-11 were phosphorylated, and these residues directly interacted with Src, suggesting a signaling pathway of claudin-11-mediated inactivation of RhoA via Src and p190RhoGAP^24^. It would be of interest to examine in future studies whether this claudin-mediated signaling occurs at TJs.

## Concluding remarks

Our understanding of the structure and function of TJs has evolved significantly in the past few years. In a widely held view, TJ formation has tended to be subordinated to the key processes in epithelial cell morphogenesis: AJ formation by cadherins and nectins along with actomyosin and cell polarity formation by polarity signaling complexes. However, functional analyses of TJ scaffolding proteins ZO-1 and ZO-2, which have been found to influence not only TJ proteins but also actomyosin and epithelial polarity, suggest that TJ formation is incorporated into the core mechanism of epithelial cell morphogenesis. This would be reasonable considering that TJ-mediated paracellular barrier formation should be coupled with polarized localizations of various transporters in the apical or basolateral plasma membrane domain for efficient epithelial transport and generation of various fluid compartments within the body. Further studies with higher resolution imaging, reconstitution experiments, and biophysical measurements will clarify the nature and dynamic aspect of TJs as super-molecular complexes that contain membrane proteins, plaque proteins, signaling molecules, and cytoskeletons.
